# Advances of 3D bioprinting technology for periodontal tissue regeneration

**DOI:** 10.1016/j.isci.2025.112532

**Published:** 2025-04-25

**Authors:** Huanhuan Chen, Yu Wang, Yue Lai, Chenda Meng, Xiner Ning, Tianmin Xu, Guangying Song, Yunfan Zhang, Yifan Lin, Bing Han

**Affiliations:** 1 Department of Orthodontics, School and Hospital of Stomatology, Peking University, Beijing 100081, China; 2 National Engineering Laboratory for Digital and Material Technology of Stomatology, Beijing Key Laboratory of Digital Stomatology, Beijing 100081, China; 3 Division of Paediatric Dentistry and Orthodontics, Faculty of Dentistry, the University of Hong Kong, Hong Kong SAR, China

**Keywords:** Applied sciences, Health sciences, Natural sciences

## Abstract

3D bioprinting technology for periodontal tissue regeneration is an advanced manufacturing technique that utilizes three-dimensional (3D) printing principles to fabricate intricate, viable structures that are specifically devised to meet with the demand for the periodontal regeneration. Personalized tissue substitutes through 3D bioprinting possesses the capability to promote tissue regeneration in tissue-defective regions. However, the current 3D bioprinting for periodontal tissue engineering still is confronted with many huge challenges that include selecting the optimal rheological properties for precise printing, ensuring the biocompatibility, and facilitating the consistent cell incorporation. In this review, we introduced the pathogenesis of periodontitis and the principle and categories of 3D bioprinting technology. We provided a comprehensive overview of the latest advancements in the application of cutting-edge biofabrication technology of 3D bioprinting for the overall periodontal regeneration, aiming to provide valuable insights for the optimization of 3D bioprinting technology and potential directions for future clinical application.

## Introduction

The periodontal tissues, comprising the gingiva, periodontal ligament, cementum, and alveolar bone, are crucial for supporting and anchoring teeth within the jawbone. Periodontal disease or injury can result in tooth loss and various other oral health issues.^1^ Given the numerous limitations of traditional treatment methods, both nonsurgical and surgical periodontal debridement often fail in fully restoring the original architecture and function of the diseased periodontal tissues, it is urgent to optimize the current regenerative treatment therapy for periodontal defect.^2^^,^^3^^,^^4^ Fortunately, the ongoing development of innovative biomaterials and therapeutic strategies holds promise for enhancing the regenerative outcomes in periodontal regeneration.^5^^,^^6^^,^^7^

It is necessary to meticulously fabricate suitable scaffolds to fully exhibit their functional capabilities according to the specific clinical contexts.^5^^,^^8^^,^^9^ For example, the high porosity and interconnectivity are beneficial for establishing an optimal microenvironment that promotes cell-to-cell communication and facilitates seamless integration of the scaffold into the adjacent tissue at the implant site.^10^ 3D bioprinting, especially utilizing bioinks that contain living cells and bioactive molecules, has emerged as a groundbreaking technology, allowing for precise and controlled modulation of porosity, interconnectivity, pore size, and permeability.^11^ This additive manufacturing technique involves the precise deposition of cell-laden hydrogels or other biomaterials in a layer-by-layer manner to create the intricate 3D structure.^12^

Several 3D bioprinting techniques have been developed, each with its own advantages and limitations. Extrusion-based bioprinting, for instance, is known for its versatility in handling a wide range of biomaterials, including those with high viscosity.^13^ However, it may face challenges in achieving high resolution and precision, which are crucial for fabricating complex tissue structures. Jetting-based bioprinting, on the other hand, offers rapid fabrication and high resolution, making it suitable for creating fine details in the scaffolds.^14^ Yet, it may be limited by the need for specific bioink properties to ensure stable jetting and cell viability. Vat photopolymerization-based bioprinting is another technique that utilizes light-induced polymerization to solidify bioinks in a layer-by-layer fashion, allowing for the creation of scaffolds with high precision and complex geometries.^15^ However, this method may be constrained by the limited selection of photocurable biomaterials and the potential for phototoxicity to cells. In summary, it is essential to select appropriate 3D bioprinting techniques that can accurately simulate the intricate structural and functional characteristics of periodontal tissues, ensuring that the scaffolds can remodel the regenerative microenvironment and promote efficient periodontal regeneration.^16^

Recent advancements in 3D bioprinting technologies have significantly improved the performance of fabricated scaffolds,^17^ including the precise positioning of cells and matrix through the use of diverse cell types and materials in bioinks.^18^ Additionally, it offers rapid fabrication, high resolution, and automation. These innovations facilitate the creation of scaffolds with tailored mechanical properties, precise degradation rates, and the ability to release therapeutic agents in a controlled manner, ultimately enhancing regenerative outcomes.^19^^,^^20^ The review introduced the pathogenesis of periodontitis and design principle and categories of 3D bioprinting technology and provides a comprehensive overview of the recent progress and future challenges and limitations in 3D bioprinting of periodontal tissues. Additionally, it discusses potential solutions to ensure the substantial benefits of bioprinting technology for on-demand periodontal tissue regeneration.

## Pathogenesis of periodontitis

**Figure 1:**
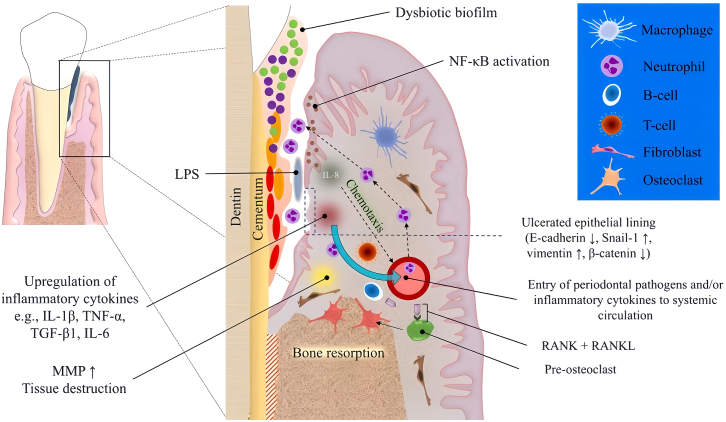
Schematic diagram of the pathogenesis of Periodontitis Reproduced with permission from ref.^25^ 2022, Saliem S S.

The periodontal tissue defects are primarily caused by periodontitis that is a chronic inflammatory disease of the periodontal tissues initiated by bacteria in the oral cavity.^2^^,^^21^^,^^22^ The intricate interplay between microbial pathogens, dysregulated host immune responses, and environmental risk factors can bring about the progressive destruction of periodontal tissues.^23^^,^^24^ Local contributing factors that disrupt this dynamic balance, such as dental calculus, tooth surface stains, anatomic defects or abnormalities of the tooth and periodontal tissues, food impaction, trauma, unhealthy habits, and poor dental restorations, can enhance bacterial accumulation and invasiveness.^25^ The pathological process of periodontitis can be roughly divided into the following steps (Figure 1).

### Bacterial colonization

The initiating factor in periodontal diseases is dental plaque biofilm, which is a soft, unmineralized bacterial community encapsulated by a matrix and adherent to the tooth surface, interdental spaces, or the surface of dental restorations. These bacteria form a highly organized architectural ecosystem that serves as the foundation for the survival, metabolism, and pathogenicity of oral bacteria. When oral hygiene is poor, dental plaque biofilm accumulates, disrupting the balance between periodontal microorganisms and the host. The microorganisms associated with periodontal diseases are primarily Gram-negative bacteria, including both obligate anaerobes (e.g., *Porphyromonas gingivalis*) and facultative anaerobes (e.g., *Aggregatibacter actinomycetemcomitans*). These periodontal pathogens selectively adhere to and colonize appropriate sites in the host, such as the tooth surface, periodontal tissues, or pre-existing plaque colonies. They then proliferate in a nutrient-rich environment, leading to the onset of periodontal diseases.

### Immune response activation

Bacterial antigens, toxins, and enzymes produced by dental plaque bacteria can directly cause periodontal tissue destruction, or indirectly contribute to it by eliciting host immune and inflammatory responses. When a dynamic equilibrium is maintained between bacterial invasion and host defense in the periodontal ecosystem, the pathogenic effects of a small number of plaque bacteria can be controlled by the host’s defense mechanisms, such as neutrophils, macrophages, lymphocytes, antibodies, and complement, thereby preserving the health of periodontal tissues.^26^ Although the host’s immune response is initially protective, aiming to prevent microbial entry into or dissemination within the periodontal tissues, some cytokines, prostaglandins, and matrix metalloproteinases (MMPs) produced during this response can mediate the destruction of periodontal connective tissue and bone. Additionally, systemic contributing factors like genetics, endocrine disorders, immune deficiencies, smoking, psychological stress, and malnutrition can weaken the host’s defense or exacerbate the inflammatory response in periodontal tissues. The onset and progression of periodontal disease also affect the pH and redox potential of the periodontal pocket, as well as the availability of oxygen and various nutrients for microorganisms, which in turn influences their growth.^27^

### Osteoclast activation

The inflammatory environment within the periodontium sets off a complex sequence of events that ultimately leads to the activation of osteoclasts, the cells primarily responsible for bone resorption. In the presence of inflammatory cytokines like TNF-α, IL-1, and IL-6, which are elevated during periodontal inflammation, osteoclast precursor cells derived from the monocyte/macrophage lineage undergo differentiation and maturation. This process involves the upregulation of transcription factors such as NFATc1 and RANKL-induced signaling pathways, which drive the expression of osteoclast-specific genes and the formation of mature osteoclasts.^22^

These activated osteoclasts then attach to the bone surface via specialized structures called podosomes, and secrete proteolytic enzymes such as cathepsin K and MMPs into the resorption pit they create. These enzymes degrade the bone matrix, particularly the organic component, allowing for the release of calcium and phosphorus ions. The osteoclasts then engulf these ions and transport them across their cell membrane, facilitating bone resorption. The overactivation of osteoclasts, fueled by the persistent inflammatory environment, results in an imbalance between bone formation and resorption, leading to the progressive destruction of alveolar bone, a crucial structural component of periodontal tissue.

### Tissue destruction

The persistent inflammatory process facilitates the destruction of other periodontal tissues, including the gingiva, periodontal ligament, and cementum. The enzymes produced by periodontal bacteria are capable of degrading nearly all cellular and extracellular components of periodontal tissues. Notably, proteases and trypsin-like enzymes that can break down collagen, fibronectin, and immunoglobulins contribute to periodontal tissue damage and attachment loss, enabling bacterial invasion into the tissues. Furthermore, these enzymes degrade tissue cellular proteins into peptides, providing a nutrient source for bacteria that lack proteolytic capabilities, thereby promoting their growth.^22^

The periodontal tissues are highly organized in a complex three-dimensional (3D) architecture, and understanding the pathogenic mechanisms of periodontitis is pivotal for facilitating the restoration or regeneration of damaged periodontal tissues.^28^ Therefore, the ultimate goal of periodontal tissue engineering is to incorporate cells, growth factors, and biomaterial to replicate the intricate and natural architecture of periodontal tissues. This endeavor is significantly aided by advancements in tissue engineering and 3D bioprinting technologies, which enable the precise fabrication of structures that closely resemble the intricate spatial organization of the native periodontal tissues.^29^

## The principle and categories of 3D bioprinting technology

### The principle of 3D bioprinting technology

**Figure 2:**
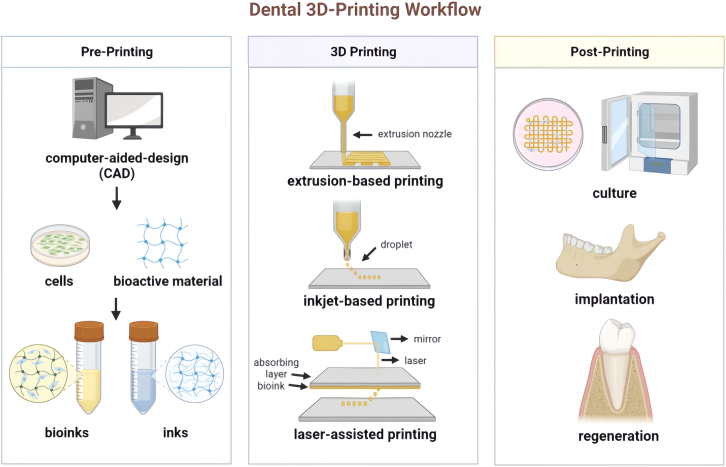
Schematic diagram of the process for 3D printing dental tissue Reprinted with permission from ref.^33^ 2024, Zhao F.

3D bioprinting is an advanced form of rapid prototyping and additive manufacturing, whereby stem cells, bioactive molecules, and biomaterial are directly printed and patterned in a layer-by-layer assembly through an automated dispensing system.^30^^,^^31^^,^^32^ It is mainly composed of three steps (Figure 2).

#### The stage of pre-printing

In the realm of 3D bioprinting, the process typically begins with the creation of a detailed blueprint using computer-aided design (CAD) modeling, which serves as the foundational plan for fabricating the intricate structure.^11^ Subsequently, a specialized 3D bioprinter is employed to meticulously deposit layers that is composed of living cells, bioactive molecules, and biomaterial scaffold, collectively termed as “bioinks”, onto a carefully prepared substrate or tissue culture dish. This deposition process is highly precise and controlled, ensuring that the resulting structure mimics the natural architecture of periodontal tissues.^34^

#### The stage of 3D bioprinting

By replicating the intricate design of these tissues, the 3D bioprinted construct is able to seamlessly integrate with the surrounding tissues and facilitate the healing process. The living cells used in this process are often encapsulated within a biodegradable scaffold material. This scaffold not only provides crucial structural support but also guides the growth of the tissues, ensuring that they develop in a controlled and organized manner.^35^^,^^36^ Additionally, bioactive molecules are incorporated into the structure, which play a pivotal role in stimulating and directing tissue regeneration, enhancing the overall efficacy of the bioprinting process.

#### The stage of post-printing

Once the 3D bioprinted construct has been successfully printed, it undergoes a period of cultivation in a laboratory setting. During this time, the cells are allowed to proliferate, differentiate, and form new tissues. This cultivation phase is essential for the maturation of the construct, ensuring that it is ready for the implantation. When the construct has reached the appropriate stage of development, it is surgically placed into the patient’s oral cavity. The seamless integration of the 3D bioprinted construct with the surrounding tissues, coupled with the stimulatory effects of the incorporated bioactive molecules, ensures a successful outcome for the patient.^33^^,^^37^^,^^38^^,^^39^

### The category of 3D bioprinting technology

3D bioprinting can be categorized into several types based on the printing mechanism and materials. The main categories include.

#### Extrusion-based 3D bioprinting

Extrusion-based bioprinting is one of the most widely used techniques in tissue engineering due to its versatility and ability to handle high-viscosity bioinks (typically ranging from 30 to 6 × 10^7^ mPa s).^13^ The printability of bioinks in this technique is influenced by several key factors, including viscosity, thixotropic properties, and shear-thinning behavior.^40^ Bioinks with optimal viscosity (typically 30–1000 mPa s) and thixotropic properties ensure smooth extrusion and shape fidelity post-printing, while shear-thinning behavior facilitates flow through the nozzle under pressure.^41^ Additionally, the crosslinking mechanism (e.g., physical, chemical, or photo-crosslinking) plays a critical role in maintaining structural integrity and printability.^42^

Cell viability in extrusion-based bioprinting is significantly affected by shear stress, material crosslinking, and nozzle diameter. High shear stress during extrusion can damage cells, particularly when using small nozzle diameters (<200 μm) or high extrusion pressures.^43^ To mitigate this, recent studies have optimized nozzle designs (e.g., conical or tapered nozzles) and reduced extrusion pressures to minimize shear-induced cell damage. The choice of crosslinking method also impacts cell viability; for instance, photo-crosslinking with biocompatible photoinitiators (e.g., lithium phenyl-2,4,6-trimethylbenzoylphosphinate or 2-hydroxy-4’-(2-hydroxyethoxy)-2-methylpropiophenone) has been shown to maintain cell viability above 90%. Typical printable cell concentrations range from 1×10^6^ to 1×10^7^ cells/mL, depending on the bioink formulation and application requirements.^44^

Despite its advantages, extrusion-based bioprinting faces limitations in resolution, with minimum feature sizes typically ranging from 200 to 1000 μm, making it challenging to replicate the intricate microstructures of biological tissues.^45^ Recent advancements have focused on improving resolution and cell viability through the development of microfluidic nozzles, advanced bioink formulations (e.g., incorporating nanoclay or graphene oxide for enhanced mechanical properties), and real-time monitoring systems.^46^ For periodontal applications, extrusion-based bioprinting has been successfully used to fabricate scaffolds with controlled porosity and mechanical properties, mimicking the complex architecture of periodontal tissues such as the periodontal ligament and alveolar bone.^47^

#### Jetting-based 3D bioprinting

Jetting-based bioprinting encompasses several techniques, including inkjet-based, laser-assisted, and laser-induced forward transfer (LIFT) bioprinting, as well as microvalve and electrohydrodynamic jetting (Table 1). These methods share the common principle of depositing bioink droplets onto a substrate with high precision, making them suitable for microscale patterning of cells and biomaterials.^14^ Briefly, inkjet-based bioprinting employs piezoelectric or thermal mechanisms to eject bioink droplets onto a substrate with high precision^48^; laser-assisted bioprinting (LAB), including laser-induced forward transfer (LIFT), utilizes a focused laser beam to transfer bioink droplets from a donor substrate to a receiver substrate with high precision^49^; microvalve bioprinting uses a mechanically controlled valve to dispense bioink droplets onto a substrate^50^; electrohydrodynamic jetting (EHD) utilizes an electric field to generate fine bioink droplets, achieving extremely high resolution (down to 1 μm).^51^

**Table 1:** Comparison of four jetting-based bioprinting techniques

Techniques	Principle	Resolution	Viscosity range	Cell viability	Advantages	Limitations	Main applications	Reference
Inkjet-based	Ejects bioink droplets using piezoelectric or thermal mechanisms	20–100 μm	3.5–12 mPa s	>90%	High speed (>10,000 droplets/s), high resolution, easy scalability	Limited bioink viscosity, prone to nozzle clogging	Cell patterning, drug delivery, tissue engineering	Liu et al. and Li et al.^48^^,^^52^
LAB/LIFT	Transfers bioink from a donor to a receiver substrate using a laser beam	10–50 μm	Up to 300 mPa s	>95%	Non-contact printing, high cell viability, no nozzle clogging	High equipment cost, strict bioink formulation requirements	Complex tissue constructs, high-resolution structure printing	Chang and Sun^49^
Microvalve	Dispenses bioink droplets using a mechanically controlled valve	50–200 μm	1–1000 mPa s	>90%	Adaptable to a wide range of bioink viscosities, multi-material printing	Lower resolution, slower printing speed	Heterogeneous tissue engineering, multi-material printing	Dudman et al.^50^
Electrohydrodynamic (EHD)	Generates fine bioink droplets using an electric field	1–10 μm	1–100 mPa s	>90%	Ultra-high resolution, suitable for micro-scale structure printing	Complex electric field control, strict bioink formulation requirements	Microvascular networks, neural tissue engineering	Reizabal et al.^51^

The printability of jetting-based bioprinting is governed by several key factors, including viscosity, surface tension, and fluid density, which can be collectively described by the Ohnesorge number (Oh).^14^^,^^53^^,^^54^ The Ohnesorge number is defined as $Oh=\frac{\eta }{\sqrt{\rho \gamma D}}$ (where η is viscosity, ρ is fluid density, γ is surface tension, and D is the nozzle diameter. For stable droplet formation, the bioink viscosity typically ranges from 3.5 to 12 mPa s, while surface tension should be optimized to ensure droplet ejection without satellite formation. Additionally, the bioink must exhibit shear-thinning behavior to facilitate flow through the nozzle while maintaining structural integrity post-deposition.^55^

Cell viability in jetting-based bioprinting is influenced by shear stress during droplet ejection, droplet impact velocity, and bioink properties.^53^ High shear stress, particularly in piezoelectric or thermal inkjet systems, can damage cells, especially when using small nozzle diameters (<50 μm). Droplet impact velocity must be controlled to minimize cell damage upon landing, typically through optimization of voltage pulses and nozzle design.^56^^,^^57^ Bioink properties, such as the presence of protective additives (e.g., alginate or Poly(ethylene oxide)-poly(propylene oxide)-poly(ethylene oxide) triblock copolymer), can further enhance cell viability by reducing mechanical stress during printing. The typical printable cell concentration for jetting-based bioprinting ranges from 1×10^6^ to 1×10^7^ cells/mL, depending on the bioink formulation and application requirements.^53^ Higher cell densities can be achieved by optimizing bioink rheology and droplet ejection parameters.^58^

Recent advancements in jetting-based bioprinting have focused on improving droplet stability, cell viability, and printing resolution. For inkjet-based bioprinting, the incorporation of shear-thinning polymers (e.g., hyaluronic acid or gelatin) into bioink formulations has enhanced droplet formation and cell survival.^52^ Multi-nozzle systems have been developed to enable simultaneous deposition of multiple bioinks, expanding the technique’s versatility for complex tissue engineering applications. In LAB/LIFT, the use of dynamic release layers and optimized laser parameters has improved printing precision and cell viability.^59^ In periodontal tissue engineering, jetting-based bioprinting has been utilized to create cell-laden constructs with precise spatial organization, facilitating the regeneration of periodontal ligament and alveolar bone.

#### Vat photopolymerization

Vat photopolymerization is a category of additive manufacturing techniques that use light to selectively polymerize photosensitive resins or bioinks layer by layer, enabling the fabrication of high-resolution 3D structures. This category includes stereolithography (SLA), digital light processing (DLP), and two-photon polymerization (2PP). SLA uses a laser to selectively cure a liquid photopolymer resin layer by layer.^60^ DLP also uses a photopolymer resin, but instead of a laser, it employs a digital light projector to cure an entire layer of resin at once.^61^^,^^62^ 2PP is a more advanced technique that uses a focused laser to induce polymerization in a very small volume of resin. Unlike SLA and DLP, which cure resin layer by layer, 2PP can create complex 3D structures by moving the laser focus point within the resin volume.^63^ These techniques are widely used in tissue engineering, dentistry, and regenerative medicine due to their ability to create complex, patient-specific structures with micron-scale precision.^15^

The printability of vat photopolymerization in bioprinting is governed by multiple interrelated factors. Central to this process are the properties of the bioink/resin, which must exhibit an optimal viscosity within 1–100 mPa s to enable precise layer deposition and high-resolution printing. Additionally, the bioink must contain photoactive groups, such as acrylates or methacrylates, to ensure efficient cross-linking upon light exposure. A critical functional requirement is shear-thinning behavior that enhances flow during printing while preserving structural integrity post-printing.^64^ Light parameters also play a pivotal role: ultraviolet (UV) light at 365 nm remains standard, though visible light systems are emerging to enhance biocompatibility. Light intensity must be carefully calibrated between 5 and 20 mW/cm^2^ to balance polymerization efficiency with cell viability, while exposure times are minimized to mere seconds per layer to mitigate UV-induced cellular damage while ensuring adequate crosslinking.^65^ Although less critical than in jetting-based techniques, the Ohnesorge number remains a secondary consideration, influencing bioink flow dynamics and layer uniformity during printing. Collectively, these parameters interact to determine the success of vat photopolymerization in bioprinting applications.

Cell viability in vat photopolymerization is influenced by three primary factors. First, photoinitiator cytotoxicity poses a critical challenge, as conventional photoinitiators like (2-hydroxy-4’-(2-hydroxyethoxy)-2-methylpropiophenone) generate cytotoxic free radicals that impair cell survival. To address this, biocompatible alternatives such as lithium phenyl-2,4,6-trimethylbenzoylphosphinate (LAP) and ruthenium-based initiators have been developed, which significantly reduce toxicity and maintain cell viability above 90%.^66^ Second, UV exposure duration directly impacts cellular health, as prolonged irradiation induces damage. Careful optimization of light intensity and exposure time is essential to minimize this risk while ensuring adequate crosslinking and structural integrity.^67^ Finally, bioink formulation plays a crucial role in enhancing cell compatibility. The incorporation of protective additives, such as alginate or (poly(ethylene oxide)-poly(propylene oxide)-poly(ethylene oxide) triblock copolymer), alongside dynamic covalent chemistry strategies, improves both cellular survival and scaffold functional performance by mitigating stress during printing and polymerization. Collectively, these factors highlight the need for a multidimensional approach to bioink design and process optimization in vat photopolymerization to achieve high cell viability and functional tissue constructs.^68^

Vat photopolymerization typically employs printable cell concentrations ranging from 1×10^6^ to 1×10^7^ cells/mL, with parameters optimized based on bioink formulations and application-specific requirements.^64^^,^^69^ Higher cell densities can be achieved through advancements in bioink rheology and light exposure calibration. Recent innovations in this field include: (1) the adoption of biocompatible photoinitiators such as lithium acylphosphinate and ruthenium-based compounds, which significantly enhance cell viability and functional preservation; (2) the integration of dynamic covalent chemistry, enabling reversible bond formation within bioinks to create scaffolds with tunable mechanical properties and improved cytocompatibility; and (3) multi-material printing strategies that combine vat photopolymerization with extrusion-based techniques, facilitating the fabrication of heterogeneous constructs with complex architectures.^67^^,^^70^ These advancements have expanded the technology’s applications in periodontal tissue engineering, particularly for creating patient-specific constructs with complex architectures and improved biological performance.^71^

In general, each of these categories has its unique advantages, making them suitable for different applications in periodontal tissue engineering. The choice of bioprinting method often depends on the specific requirements of the project, such as the type of bioink used, the desired resolution, and the complexity of the structure to be printed.

## Application of 3D bioprinting technology in periodontal tissue regeneration

To recapitulate the intricate aspects of the 3D micro-tissue environment, bioprinting can produce identical biomimetic 3D scaffolds with consistent cell distribution, thereby enabling reproducible 3D cell cultures.^11^ The bioink would comprise cells that have the potential to differentiate into periodontal tissue cells, along with carrier components that facilitate cell attachment, proliferation, and differentiation.^72^ Through distinct gelation mechanisms and compositional attributes, bioinks seamlessly align with the unique structural and regenerative requirements of this tissue. The thermally-induced gelation of bioinks enables precise temperature-controlled solidification, mirroring the need for exact placement and architecture in the intricate layers of periodontal tissue. This precision is crucial for the accurate of the tissue’s complex structure, which is vital for its function and regeneration.^46^ On the other hand, chemically-induced gelation, triggered by agents like cross-linkers or pH changes, offers a dynamic response that can be tailored to mimic the biological cues driving periodontal tissue formation and repair. Furthermore, the composition of bioinks plays a pivotal role in addressing the specific requirements of periodontal tissue regeneration. Cell-based bioinks, enriched with stem cells, progenitor cells, or periodontal-derived cells, directly promote cell differentiation and tissue regeneration. This cellular composition is essential for recapitulating the native cellular environment of periodontal tissue, fostering the growth and organization of cells into functional structures. In contrast, scaffold-based bioinks provide a supportive framework with biocompatible polymers or hydrogels, akin to the natural extracellular matrix (ECM) of periodontal tissue. These scaffolds, whether derived from natural sources such as collagen or hyaluronic acid, or synthetic materials like polyethylene glycol or pluronic, significantly impact the mechanical integrity, biodegradability, and biocompatibility of the 3D bioprinted tissue constructs. The careful selection of scaffold materials ensures that the printed structures not only mimic the physical properties of native periodontal tissue but also support cell attachment, growth, and the eventual formation of a functional, regenerative tissue. Thus, the synergy between 3D bioprinting technology and the inherent characteristics of bioinks offers a promising approach for the precise, controlled, and biologically inspired regeneration of periodontal tissue.

### Natural scaffolds-based bioinks for 3D bioprinting technology

Natural scaffolds are extensively utilized in periodontal tissue engineering due to their outstanding biocompatibility and biodegradability (Table 2). These scaffolds inherently possess bioactive qualities, enabling them to interact actively with cellular components, fostering a microenvironment that is conducive to tissue regeneration and healing.

**Table 2:** Natural scaffolds-based bioinks for 3D bioprinting technology

Components	Cell source	Bioprinting type	Function	Reference
Type 1 collagen	Periodontal ligament cell	Extrusion-based 3D bioprinter	Develop a biomimetic microfibrous system with the capability to endure functional loads and facilitate periodontal ligament regeneration	Lin et al.^47^
Type I lyophilized collagen	Periodontal ligament stem cells (PDLSCs)	Extrusion-based 3D bioprinter	Explore a collagen-based bioink that mimics native ECM conditions and delivers PDLSCs to direct periodontal ligament organization and regeneration	Araujo et al.^73^
Acellular dermal matrix (ADM)/gelatin/sodium alginate	Gingival fibroblast cells (GFs)	3D bioprinter	Assess ADM’s efficacy *in vivo* for keratinized gingiva augmentation, exploring its clinical potential in periodontal tissue regeneration.	Liu et al.^74^
Gelatin methacryloyl (GelMA) hydrogel precursor	Periodontal ligament cell	Microextrusion-based 3D bioprinter	Determine the optimal printing conditions for achieving high resolution, dimensional stability, and cell viability in 3D bioprinting of periodontal ligament cells	Thattaruparambil Raveendran et al.^75^
Gelatin methacrylate (GelMA)	Hertwig’s epithelial root sheath (HERS) cells and dental papilla cells (DPCs)	Extrusion-based 3D bioprinter	Enhance epithelial-mesenchymal interaction (EMI) to foster alveolar bone regeneration	Tang et al.^76^
Decellularized extracellular matrix (dECM)	Human bone marrow mesenchymal stem cells (hBMSCs)	3D bioprinter	Illustrate the cellular-level impacts of compositional variations on tissue specificity; analyze tissue-specific patterns of differential gene expression	Han et al.^77^
Methacrylate gelatin/decellularized extracellular matrix (GelMA/dECM)	Dental follicle cells (DFCs)	Digital light projection (DLP)-based 3D bioprinter	Solve periodontal ligament orientation and interface reconstruction issues in periodontal complex design; develop a porcine dental follicle-derived dECM bioink for better periodontal regeneration	Yang et al.^78^

#### Collagen-based scaffold

Collagen, a protein abundantly found in human organ tissues, possesses remarkable properties that enable cells to recognize and adhere to it with exceptional efficiency. Specifically, collagen-based materials have emerged as highly promising biogenic bioinks for the regeneration of various tissues, distinguished by their exceptional cell-activating capabilities and biocompatibility.^79^ Lin et al.^47^ have successfully fabricated collagen-based straight and waveform microfibers using an extrusion-based bioprinter to direct the growth of periodontal ligament cells. Additionally, they employed a laminar flow-based bioreactor to induce fluidic shear stress. The findings indicated that the 3D bioprinted collagen-based waveform microfibers not only maintained periodontal ligament cell viability but also demonstrated an increased propensity to facilitate healing and regeneration when subjected to shear stress. Araújo et al.^73^ developed a collagen-based bioink that mimics the natural ECM and contains periodontal ligament stem cells (PDLSCs) to direct periodontal ligament formation. The bioink showed good biocompatibility *in vivo*, with PDLSC alignment, organization, and migration to the root surface observed in bioprinted scaffolds. This versatile bioink supports PDLSC proliferation and differentiation, offering potential for bioprinting scaffolds for periodontal ligament regeneration.

Acellular dermal matrix (ADM), an innovative biomaterial mainly composed of collagen, possesses a unique combination of structural and functional properties that make it highly suitable for various medical and surgical applications. This advanced material is characterized by its porous structure and exceptional biocompatibility, rendering it a valuable asset in tissue engineering and wound healing.^80^^,^^81^ Herein, Liu et al.^74^ created 3D bioprinted ADM scaffolds encapsulating gingival fibroblasts (GFs) and evaluated their efficacy in augmenting keratinized gingiva *in vivo*, which showed superior tissue integration and elevated levels of collagen and Vascular Endothelial Growth Factor A (VEGF-A) in the GF-infused group.

#### Gelatin-based scaffold

Derived from collagen through acid or alkaline hydrolysis, gelatin exhibits contrasting gelation behavior compared to collagen, while demonstrating excellent cytocompatibility, hydrophilicity, and adequate degradability. Gelatin methacryloyl (GelMA) is produced by modifying the amino acids of gelatin with methacryloyl groups. This modification endows GelMA with both the natural characteristics of gelatin and the controllable properties of photosensitive polymers, making it an ideal bioink for 3D bioprinting applications. Raveendran et al.^75^ have conducted a systematic investigation into the printability of GelMA hydrogel precursor at various concentrations using a microextrusion-based 3D bioprinter. Through this comprehensive evaluation, they were able to identify the optimal printing conditions that ensure high printing resolution, dimensional stability, and cell viability for the 3D bioprinting of periodontal ligament cells. The epithelial-mesenchymal interaction (EMI) plays a pivotal role in bone regeneration. Tang et al. utilized 3D bioprinting to recombine Hertwig’s epithelial root sheath (HERS) cells with dental papilla cells (DPCs), embedding them in GelMA to mimic the *in vivo* microenvironment of cell-cell interactions, which provided an optimal environment for HERS cells and DPCs to produce mineralization textures and facilitate alveolar bone regeneration via their interactions.^76^

#### Decellularized extracellular matrix (dECM)-based scaffold

**Figure 3:**
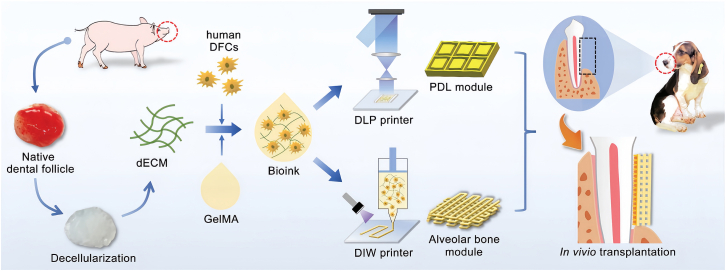
Schematic overview of the 3D bioprinting of periodontal modules with GelMA/dECM bioink encapsulating human DFCs for periodontal regeneration Reproduced with permission from ref.^78^ 2023, Yang X.

The dECM of tissues/organs is a naturally sourced biomaterial that retains diverse functional components from the original tissue or organ. The integration of dECM methodologies with 3D bioprinting has led to substantial advancements in the development of highly biomimetic and reliable tissue/organ platforms, marking significant strides in the field of tissue engineering.^19^ Han et al. conducted comprehensive differential proteomic analyses on four types of porcine dECMs. Additionally, they performed microarray analyses on human bone marrow mesenchymal stem cells (hBMSCs) printed with various dECM bioinks to elucidate the tissue-specific effects of compositional variations at the cellular level, taking into account the multipotency of MSCs.^77^ While 3D bioprinting with dECM bioink has shown promise in various tissues, the limited availability of periodontal ligament tissue for dECM production poses a challenge. Dental follicle tissue, as a precursor to periodontal structures, offers a more abundant and suitable source for dECM bioink, making it a promising candidate for periodontal regeneration through 3D bioprinting.^82^ Yang et al. developed a dental follicle-derived dECM bioink and combined it with methacrylate gelatin to create a methacrylate gelatin/decellularized extracellular matrix (GelMA/dECM) cell-laden bioink for 3D bioprinting (Figure 3), which displayed excellent immunomodulatory activity and significantly enhanced periodontal tissue regeneration in a clinically relevant animal model.^78^

### Synthetic scaffolds-based bioinks for 3D bioprinting technology

Synthetic biomaterials, encompassing bioceramics, synthetic polymers, metals, and alloys, have gained extensive utilization in 3D bioprinting applications owing to their exceptional mechanical properties and formability (Table 3). Given the inferior mechanical properties of natural scaffolds, which undermine their reliability in the printing process and their ability to regenerate hard tissues such as bone, a bioink that combines natural and synthetic biomaterials displayed superior outcomes both *in vitro* and *in vivo*, compared to a bioink composed solely of natural biomaterials.^72^

**Table 3:** Synthetic scaffolds-based bioinks for 3D bioprinting technology

Components	Cell source	Bioprinting	Function	Reference
Collagen/strontium-doped calcium silicate (SrCS)	Gingival fibroblast cell (GFs)	Multi-extrusion-based 3D bioprinter	Evaluates the 3D printed SrCS scaffold’s potential in future periodontal engineering and clinical applications	Wang et al.^83^
Gelatin methacryloyl (GelMA)/sodium alginate (SA)/bioactive glass microsphere (BGM)	Mouse bone marrow mesenchymal stem cells (mBMSCs)	Extrusion-based 3D bioprinter	Develop an innovative multi-component hydrogel that exhibits excellent biological activity, enabling it to encapsulate viable cells for 3D bioprinting and promote the regeneration of both soft and hard periodontal tissues	Miao et al.^84^
Alginate/gelatin/nano-hydroxyapatite (AGH)	Gingival fibroblast cells (GFs); bone marrow mesenchymal stem cells (BMSCs)	Extrusion-based 3D bioprinter	Develop a 3D cell-printed soft-hard construct utilizing two distinct bioinks for socket healing, and assess its biocompatibility	Zhang et al.^85^
Sodium alginate (SA)/gelatin (Gel)/nano-hydroxyapatite (na-HA)	Human periodontal ligament stem cells (hPDLSCs)	Extrusion-based 3D bioprinter	Provide a foundational experimental basis for further investigating the use of 3D bioprinting to create bioscaffolds for bone defect reconstruction	Tian et al.^86^
Alginate/polyvinyl alcohol (PVA)/hydroxyapatite	Mouse calvarial 3T3-E1 (MC3T3) cells	Extrusion-based 3D bioprinter	Explore the utilization of 3D printing technology with alginate hydrogels to fabricate biocompatible and osteoconductive scaffolds tailored for bone defect repair	Bendtsen et al.^87^
Polycaprolactone (PCL)/Sr-doped nano hydroxyapatite (Sr-nHA)	Human osteosarcoma cells	Extrusion-based 3D bioprinter	Refine the intricate structure and functionality of the periodontal tissue	Porta et al.^88^
Polycaprolactone-hydroxyapatite (90:10wt %)	Dental pulp stem cells (DPSCs)	3D bioprinter	Develop multiphase region-specific microscaffolds using three-dimensional printing for integrated periodontium regeneration	Lee et al.^89^
Gelatin methacrylate (GelMA)/Polyethylene glycol diacrylate (PEGDA)	Periodontal ligament stem cells (PDLSCs)	3D bioprinter	Develop a bioprinting strategy to investigate stem cell-ECM interactions and identify a suitable ECM for *in vivo* alveolar bone defect repair	Ma et al.^90^
Polyethylene glycol diacrylate (PEGDA)/Pluronic F127 diacrylate (F127DA)/gelatin methacrylate (GelMA)	Rabbit bone marrow mesenchymal stem cells (rBMSCs)	Digital light projection (DLP)-based 3D bioprinter	Explore the GelMA/PEGDA/F127DA (GPF) scaffold’s potential for treatment of bone defects	Gao et al.^91^
Glycidyl methacrylate (GMA) modified epsilon-poly-L-lysine (EPLGMA)	Periodontal ligament stem cells (PDLSCs)	Digital light projection (DLP)-based 3D bioprinter	Develop a multifunctional and biomimetic alveolar bone module for periodontitis-derived bone defect repair	Yu et al.^92^
Hyperelastic bone (HB, 90%weight (wt) hydroxyapatite and 10%wt poly(lactic-co-glycolic acid))/superparamagnetic iron oxide nanoparticles (SPIONs)	Embryonic murine C3H10T12 cells and human-patient-derived osteoblast-like HBO cells	3D bioprinter	Assess the osteoregenerative capacity of bioprinted scaffolds both *in vitro* and *in vivo* in a critical-sized bone defect in the rat femur	Shokouhimehr et al.^93^
Methacrylated gelatin (GelMA)/methacrylated alginate (AlgMA)/laponite (Lap)	Rat bone marrow mesenchymal stem cells (BMSCs)	3D bioprinter	Development a bioink with rapid internal vascularization capabilities and relatively sustained osteoinductive bioactivity	Cao et al.^94^

#### Bioceramics-based scaffolds

Numerous studies have verified that calcium silicate (CS) fosters the development of a hydroxyapatite layer on scaffolds, thereby strengthening the bond between scaffolds and surrounding bone tissues, whereas strontium (Sr) not only prevents fractures related to osteoporosis but also stimulates collagen synthesis to promote bone tissue formation. Wang et al. created a 3D bioprinted bi-layer scaffold that integrates guided tissue regeneration (GTR) with a collagen/strontium-doped calcium silicate (SrCS) composition for the periodontal regeneration. Both *in vitro* and *in vivo* studies revealed improved bone formation and increased osteogenic protein secretion, suggesting its promising potential for clinical use in periodontal and bone regeneration.^83^

**Figure 4:**
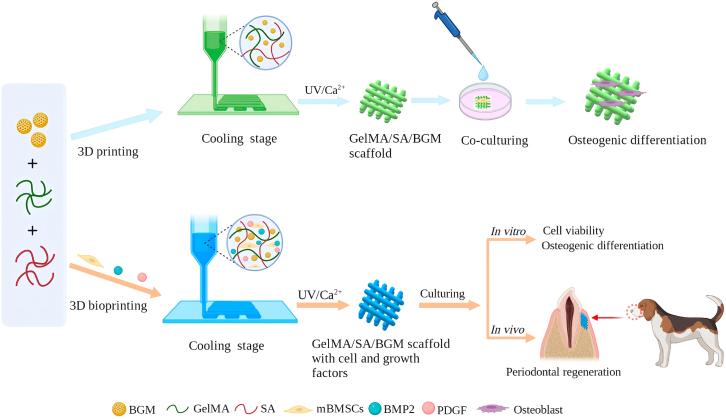
Flowchart of fabrication and characterization of 3D bioprinted GelMA/SA/BGM scaffold and 3D bioprinted cell- and growth factor-laden GelMA/SA/BGM scaffold Reproduced with permission from ref.^84^ 2023, Miao G.

To develop multi-component bioink with excellent biological activity that can encapsulate living cells for 3D bioprinting and facilitate the regeneration of both periodontal soft and hard tissues, GelMA and alginate (Algin) can be integrated with bioceramics to improve the poor mechanical properties.^95^^,^^96^ Miao et al. developed a novel multi-component hydrogel consisting of GelMA, sodium alginate (SA), and bioactive glass microspheres (BGM) (Figure 4). The BGM-loaded scaffolds demonstrated good biocompatibility, osteogenic differentiation, apatite formation, and mechanical strength. *In vivo* studies in Beagle dogs showed significant periodontal tissue regeneration, including gingival tissue, periodontal ligament, and alveolar bone.^84^ Zhang et al. developed a 3D cell-printed soft-hard construct composed of alginate/gelatin (AG) with GFs and alginate/gelatin/nano-hydroxyapatite (AGH) with BMSCs. *In vivo* studies showed better healing and integration of the cellular printed construct compared to acellular constructs, which display huge potential for alveolar ridge preservation after tooth extraction, providing a customized plug to minimize bone resorption and improve gingival growth.^85^ Additionally, Tian et al. developed a hydrogel by mixing sodium alginate (SA), gelatin (Gel), and nano-hydroxyapatite (na-HA), and determined the best printing slurry based on its rheological properties. By integrating human periodontal ligament stem cells (hPDLSCs) into the printing material, they created SA/Gel/na-HA/hPDLSCs cell bioscaffolds. These bioscaffolds effectively promoted cell survival, proliferation, and osteoblast differentiation, suggesting their potential for bone defect repair.^86^ Bendtsen et al. devised a novel hydrogel composition that blends alginate, PVA, and HA, specifically designed for 3D bioprinting of mouse calvarial 3T3-E1 (MC3T3-E1) cells to create high fidelity scaffolds. This cutting-edge, osteoconductive, and biodegradable alginate-PVA-HA formulation, along with its proficiency in 3D bioprinting tissue-engineered scaffolds, offers promising potential for the personalized treatment of bone defects.^87^

#### Synthetic polymers-based scaffold

Polycaprolactone (PCL) is a biodegradable and biocompatible polyester that has been explored for various medical and dental applications. It can be used to create scaffolds or membranes that provide a supportive structure for the growth and differentiation of cells involved in periodontal tissue regeneration, such as fibroblasts, osteoblasts, and cementoblasts. These scaffolds can help guide the formation of new bone, cementum, and periodontal ligament, thereby promoting the regeneration of the periodontal tissues.^97^^,^^98^ Porta et al. fabricated a porous multi-layer scaffold using PCL and Sr-doped nano hydroxyapatite (Sr-nHA) via a single-step extrusion process. The multi-layer scaffold showed improved biological, mechanical, and osteogenic capabilities compared to single-layer scaffolds. *In vitro* studies indicated that the presence of ceramics in the polymeric matrix enhanced bone mineralization-related protein secretion. The single-step process ensured excellent integrity between the scaffold layers.^88^ To develop multiphase region-specific microscaffolds with spatiotemporal delivery of bioactive cues for integrated periodontium regeneration, Lee et al. created polycaprolactone-hydroxyapatite (90:10 wt %) scaffolds utilizing seamless 3D bioprinting in three distinct phases. These scaffolds were meticulously designed with varying microchannel sizes to replicate the intricate structures of the cementum/dentin interface, periodontal ligament, and alveolar bone. This innovative approach highlights the potential for regenerating complex multiphase periodontal tissues through the spatiotemporal delivery of multiple proteins, guiding a single stem/progenitor cell population to differentiate into the diverse tissues of the periodontium.^89^

The GelMA can also be integrated with synthetic polymers to enhance its performance and versatility. For example, polyethylene glycol diacrylate (PEGDA), a derivative of polyethylene glycol (PEG), is a promising synthetic polymer with excellent biocompatibility, printability, and mechanical properties.^99^ Ma et al. utilized a 3D bioprinting platform to fabricate hydrogels with varying compositions of PDLSC-laden GelMA/PEGDA. The 4:1 GelMA/PEGDA ratio exhibited optimal osteogenic differentiation *in vitro*. *In vivo* studies revealed significantly enhanced bone formation with the optimized PDLSC-laden hydrogel compared to control groups. This innovative approach holds promise for advancing functional tissue regeneration.^90^ Additionally, Gao et al. developed a sophisticated bone tissue engineering (BTE) scaffold utilizing DLP printing technology, incorporating polyethylene glycol diacrylate (PEGDA), Pluronic F127 diacrylate (F127DA), and GelMA. This scaffold significantly stimulates osteogenic differentiation of MSCs within an osteoinductive milieu, presenting a novel strategy for addressing bone defects.^91^

Biomaterials used for alveolar bone regeneration ideally possess antibacterial properties to guard against infection-related complications.^100^ The bioink that is formulated with glycidyl methacrylate (GMA) modified EPL (EPLGMA) hydrogels has demonstrated effective antibacterial capabilities. Herein, Yu et al. designed a 3D bioprinting module utilizing a bioink composed of glycidyl methacrylate (GMA) modified epsilon-poly-L-lysine (EPLGMA), loaded with PDLSCs and myeloid-derived suppressive cells membrane vesicles (MDSCs-MVs) for repairing bone defects caused by periodontitis. It provided an optimal microenvironment for bone regeneration and exhibited effective antibacterial activity against periodontopathic bacteria. This multifunctional 3D bioprinting bioink presents promising new opportunities for bone defect repair and clinical applications.^92^ However, in cases of non-infection-related bone defects, such as large bone fractures, many clinical products and their surgical applications face significant technical, surgical, and manufacturing/scaling challenges, and the regeneration of such fractures often achieves limited clinical success, primarily due to poor integration and healing.^101^

In this context, hyperelastic Bone (HB) stands out as a highly promising osteoregenerative bioink. With the incorporation of polylactic-co-glycolic acid (PLGA) polymer, this cutting-edge biomaterial boasts up to 90% hydroxyapatite content and demonstrates remarkable elastic properties.^102^ Shokouhimehr et al. 3D bioprinted a new generation of hyperelastic bone (HB) implants loaded with superparamagnetic iron oxide nanoparticles (SPIONs) and studied their effect on large non-healing bone fractures. The addition of SPIONs enhances the bacteriostatic properties of the bone grafts without causing cytotoxicity. *In vitro* tests show that cells remain viable on the printed scaffolds, and implantation in a rat model demonstrates significant bone regeneration over 2 weeks, with rapid integration, ossification, and new bone growth. These findings suggest promising potential for 3D bioprinted HB scaffolds in hard tissue engineering.^93^

#### Nanoclays-based scaffold

Nanoclays, such as laponite (Lap), have emerged as a novel category of biocompatible materials that exhibit robust drug loading capabilities and hold promise as strength-enhancing additives.^103^ Incorporating Lap into hydrogels can influence both the mechanical properties and the immune microenvironment of bioinks. Cao et al. incorporated rat platelet-rich plasma (PRP) into a GelMA/AlgMA hydrogel system modified with Lap to create a PRP-GA@Lap hydrogel. This hydrogel sustained the release of growth factors for up to 2 weeks and significantly promoted stem cell proliferation, migration, and osteogenic differentiation *in vitro*. When combined with PCL to form 3D bioprinted scaffolds, the PRP-GA@Lap/PCL scaffolds significantly enhanced vascular growth and bone regeneration in rat models. This study highlights the potential of PRP-based 3D bioprinted scaffolds for bone defect treatment.^94^

## Significance and future challenge in 3D bioprinting for periodontal tissue regeneration: Harnessing machine learning for optimization and personalized solutions

Essentially, the remarkable advancement of 3D bioprinting ensures the cohesive dispensing of individual and multiple cell types within biocompatible materials, facilitating the creation of desired 3D functional structures.^104^^,^^105^ The role of 3D bioprinting in periodontal treatment is multifaceted and significant. It primarily includes.

(1) Enabling personalized treatment solutions: 3D bioprinting technology allows for the creation of customized scaffolds or implants that precisely match the patient’s specific anatomical needs and defects in the periodontal tissues. This personalized approach enhances the accuracy and effectiveness of the treatment.^11^
(2) Promoting tissue regeneration: By mimicking the complex 3D structures of periodontal tissues, 3D bioprinting can produce scaffolds with optimal mechanical, rheological, and biological properties that support cell adhesion, proliferation, and differentiation. This fosters the regeneration of periodontal tissues, including the gingiva, alveolar bone, cementum, and periodontal ligament.^17^
(3) Addressing complex regeneration challenges: Traditional periodontal treatments often face challenges such as graft material retention, membrane stability, infection control, and blood supply. 3D bioprinting technology addresses these issues by providing precisely designed and tailored solutions that optimize healing conditions and enhance the overall success of the regenerative process.^19^^,^^29^
(4) Accelerating research and development: The advancements in 3D bioprinting facilitate rapid prototyping and testing of new materials, designs, and therapeutic strategies for periodontal tissue regeneration. This accelerates the pace of research and development, leading to more effective and efficient treatments for patients.^104^^,^^106^
(5) Improving clinical outcomes: By enabling the precise reconstruction of periodontal tissues, 3D bioprinting technology has the potential to significantly improve clinical outcomes. Patients can experience faster healing, reduced discomfort, and better functional and aesthetic results after periodontal treatment.^33^

**Figure 5:**
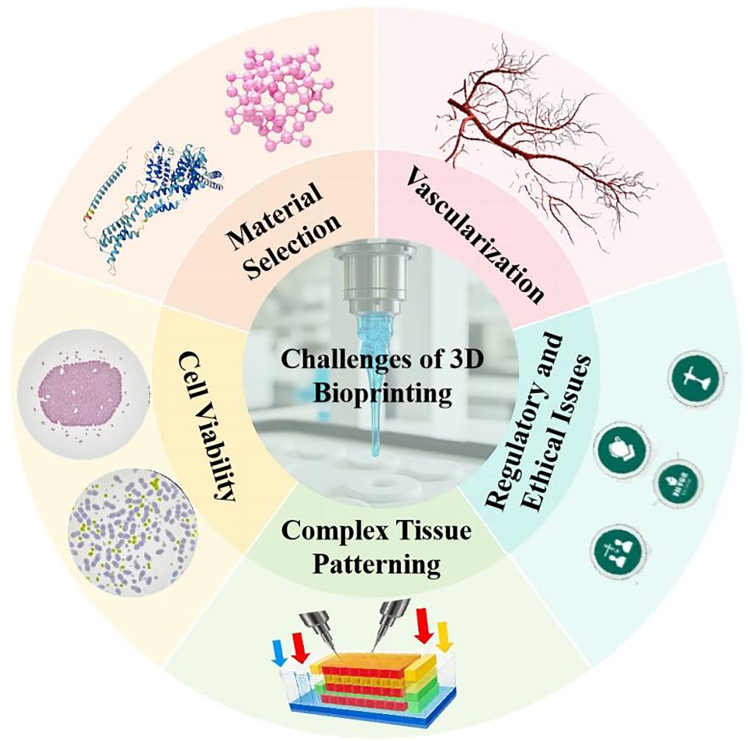
Challenges of 3D bioprinting in periodontal tissue regeneration

Despite being a promising technology for periodontal tissue regeneration, 3D bioprinting faces several significant challenges^106^^,^^107^ (Figure 5). First, the identification of suitable biodegradable and biomimetic printable materials that can promptly support cell attachment and proliferation is crucial. The “bioinks” required for 3D bioprinting must possess excellent biocompatibility and mechanical properties to mimic the natural ECM and facilitate cellular activities.^108^ However, the selection of such materials remains limited, particularly those that can effectively regenerate the complex periodontal tissue comprising gingiva, alveolar bone, cementum, and periodontal ligament. Second, the need for vascularization at the single-cell level poses a major hurdle. Periodontal tissues are highly vascularized, and the regeneration process requires the formation of a functional vascular network to supply nutrients and oxygen to the newly formed tissues. Current 3D bioprinting technologies struggle to achieve this level of vascularization, leading to potential necrosis and non-viability of the printed constructs.^109^ Third, the complex patterning of heterocellular tissues is another challenge. The periodontal tissue is composed of multiple cell types, each with specific functions and interactions. 3D bioprinting must precisely control the spatial distribution and organization of these different cell types to mimic the native tissue architecture.^33^ This requires advanced biofabrication techniques and precise control over the printing process.

Moreover, maintaining cell viability and long-term functionality until tissue regeneration or remodeling is essential. The printing process itself can be detrimental to cell health due to shear stress, temperature changes, and exposure to non-physiological conditions. Ensuring high cell survival rates and sustained functionality throughout the regeneration process is critical but remains a significant challenge.^17^^,^^104^ Lastly, the translation of 3D bioprinting technologies from the laboratory to the clinic faces ethical and regulatory issues. The use of human cells in bioprinting raises concerns about consent, privacy, and potential misuse. The regulatory landscape for 3D bioprinted medical products is still evolving, and clear guidelines and standards are needed to ensure safety and efficacy.^110^

In summary, 3D bioprinting in periodontal tissue regeneration faces multifaceted challenges, including material selection, vascularization, complex tissue patterning, cell viability, and regulatory hurdles. However, with the rapid development of new technologies, recent advancements in machine learning (ML) have shown great promise in addressing these challenges by optimizing the bioprinting process and material development, particularly for complex applications such as periodontal tissue regeneration.^111^ ML techniques offer a powerful approach to address key challenges in bioprinting, such as printability, cell viability, and material properties, by leveraging data-driven methodologies.^112^

The bioprinting process is governed by multiple interdependent parameters (e.g., nozzle pressure, printing speed, layer thickness, light exposure time) that significantly affect print quality and cell viability. ML algorithms, such as neural networks and genetic algorithms, can analyze extensive datasets derived from experimental trials to identify optimal parameter combinations. For instance, supervised learning models can establish predictive relationships between input parameters (e.g., bioink viscosity, crosslinking time) and output metrics (e.g., printing resolution, cell survival rate), thereby enabling more efficient and precise optimization of the bioprinting process.^113^ Additionally, ML can accelerate the discovery and formulation of novel bioinks by predicting material properties based on chemical composition and processing conditions. For instance, reinforcement learning can guide the selection of polymers, crosslinkers, and cell types to achieve desired mechanical strength and biological functionality. Xu et al.^114^ have utilized a ML framework to accurately predict the viscosity of heterogeneous bioink compositions to enhance extrusion-based bioprinting techniques.

Post-printing tissue maturation and integration with native tissues are critical for successful periodontal regeneration. To address this, ML models can predict the long-term behavior of bioprinted constructs, such as cell differentiation, ECM deposition, and vascularization. For example, time-series analysis using recurrent neural networks (RNNs) can simulate the maturation process and guide the design of bioprinted scaffolds.^115^ Furthermore, periodontal tissue regeneration requires personalized approaches due to variations in patient anatomy and disease severity. In this context, ML can integrate patient-specific data (e.g., CT/MRI scans and genetic information) to design customized bioprinted scaffolds and optimize treatment strategies. However, despite its significant potential, challenges remain, such as the need for high-quality datasets, computational costs, and the integration of ML models with experimental validation. Therefore, future research should focus on developing robust ML frameworks tailored to the specific needs of periodontal tissue regeneration.^116^

## Conclusions and perspectives

Periodontitis and severe trauma act as major causes for the destruction to the periodontal ligament. The magnitude and configuration of the periodontal defect from each patient is totally different. Repairing the native and integral conditions of the periodontal ligament is essential for the stability and function of the tissue. The realm of 3D bioprinting technology for periodontal tissue regeneration opens up new avenues for treating periodontal diseases and improving oral health. This technology, by virtue of its precision and versatility, is poised to revolutionize the landscape of periodontal regeneration. One of the most compelling aspects of 3D bioprinting lies in its ability to precisely control the shape, size, and composition of the regenerated tissues. This level of customization ensures that each patient receives a tailored solution, addressing their unique anatomical and physiological needs.^11^^,^^104^ This is a marked improvement over traditional regenerative therapies that often rely on one-size-fits-all approaches. The use of 3D imaging and CAD software facilitates the creation of highly accurate scaffolds that mimic the complex architecture of native periodontal tissues.

Moreover, the incorporation of stem cells and bioactive molecules within the scaffolds amplifies the regenerative potential of 3D bioprinting. These cells that are induced by various kinds of molecules can differentiate into various periodontal cell types, promoting the formation of new cementum, periodontal ligament, and alveolar bone. The controlled release of growth factors and cytokines from the scaffolds further enhances tissue regeneration by modulating the immune response and stimulating angiogenesis.^17^^,^^106^

The development of innovative biomaterials and bioinks is also crucial for the success of 3D bioprinting in periodontal tissue regeneration. These materials must not only be biocompatible and biodegradable but also possess the appropriate mechanical properties to support tissue growth and function. Natural materials such as collagen, hyaluronic acid, and chitosan, as well as synthetic polymers like polylactic acid (PLA) and polyglycolic acid (PGA), have shown great promise in this regard. Further optimization of these materials, as well as the exploration of novel bioinks, is essential for optimizing the current technology and solving some problems.^117^

Despite these advancements, it still faces many challenges in replicating the complex architecture and functional properties of periodontal tissues. The periodontal complex is a highly organized structure with specific orientations and arrangements of different tissue types. Achieving this level of organization through 3D bioprinting is relatively difficult. Recent research has showed 4D, 5D, and 6D bioprinting techniques may offer additional capabilities such as shape-shifting and the integration of multiple materials with distinct properties, paving the way for the creation of more sophisticated tissue constructs.^118^ ML has emerged as a powerful tool to address the challenges associated with these advanced bioprinting techniques, particularly in optimizing the bioprinting process and material development for complex applications like periodontal tissue regeneration. As the field continues to evolve, future research should focus on overcoming remaining challenges, such as improving the long-term biocompatibility and functionality of scaffolds, enhancing cost-effectiveness, and increasing accessibility to a broader patient population. Integrating ML-driven approaches into these efforts will be critical to accelerating progress and achieving clinically viable solutions.

In summary, the advancements in 3D bioprinting technology for periodontal tissue regeneration hold great potential in dentistry. By addressing these challenges associated with biomaterial development, scaffold design, and tissue organization, this technology can revolutionize the treatment of periodontal diseases and make great contributions to the broader regenerative medicine field. With further research and development, it is promising for the scientists and clinicians to embrace a new era of personalized and precise dental regenerative therapies.

## Acknowledgments

This work was funded by the 10.13039/501100001809National Natural Science Foundation of China (52473120, U21A2055, 82201017, 82201020, 82071172), Beijing Natural Science Foundation (L242037, L242133), Young Elite Scientist Sponsorship Program by CAST (2022QNRC001), 10.13039/501100002858China Postdoctoral Science Foundation (2022M710257), Ningxia Hui Autonomous Region key Research and Development program (2022BEG02031), 10.13039/501100005847Health and Medical Research Fund (No. 19201421), National clinical key discipline construction project (PKUSSNKT-T202102).

## Author contributions

Conceptualization, B.H., Yifan Lin, and Y.Z.; writing–original draft preparation, H.C. and Y.W.; writing–review and editing, H.C. and Y.W.; visualization, B.H., Yifan Lin, and Y.Z.; supervision, Yue Lai, C.M., X.N., T.X., and G.S.; funding acquisition, B.H., Yifan Lin, Y.Z., G.S., T.X., and Y.W. All authors have read and agreed to the published version of the manuscript.

## Declaration of interests

The authors declare no conflicts of interest.
